# Australian podiatrists' understanding and current practices of reablement for older people: a qualitative exploratory study

**DOI:** 10.1186/s13047-022-00578-9

**Published:** 2022-11-08

**Authors:** Lachlan Mulquiny, Jodi Oakman

**Affiliations:** Centre for Ergonomics and Human Factors, School of Psychology and Public Health, La Trobe University, Melbourne, VIC Australia

**Keywords:** Podiatry practice, Aged care, Reablement, Qualitative

## Abstract

**Background:**

The ageing of Australia's population is placing significant pressure on health and social aged care services due to increasing demand for the provision and a relative decrease in the healthcare workforce. Reablement has been introduced by the Australian Commonwealth Government and is aimed at increasing older people's independence to age in place and decreasing dependency on aged care services. To date, research on reablement practice has focussed on interventions from physiotherapists, occupational therapists, and nurses, with no data available on podiatrist involvement. The aim of this research was to explore Australian podiatrists' understanding and current practice of implementing a reablement approach to older clients.

**Methods:**

A qualitative exploratory study was conducted with Australian podiatrists who had experience working with older people and were familiar with the reablement model. Podiatrists were recruited after completing a prior web-based survey. Promotion of the web-based survey was via professional networks and Twitter. Interviews were audio-recorded, transcribed verbatim, and analysed using Braun and Clarke's approach to thematic analysis.

**Results:**

Fourteen podiatrists were interviewed. Using thematic analysis, three themes were generated: (i) Thinking and practicing differently, (ii) Reconciling practice with competing pressures, (ii) Funding influences on podiatry practice and reablement. Rather than identifying practice examples that demonstrate involvement by podiatrists in older peoples reablement, our analysis identified system level barriers which gave negative influence on podiatrists' ability to implement the reablement model.

**Conclusions:**

The participants in this study considered their role in reablement for older people was limited. While some participants felt unskilled to implement the reablement model, it is factors such as inadequate funding arrangements and clients' perceptions of podiatrists' roles have a more significant impact on current practice and are seemingly more intractable.

## Background

The ageing population is placing significant challenges on health and social aged care services in Australia [1, 2]. In 2011, the proportion of Australia's population aged 65 years and over was 14.6% and is predicted to rise to 19.4% by 2031. Current predictions estimate an increase in government expenditure on aged care from 0.8% of gross domestic product (GDP) in 2009–10 to 1.8% of GDP by the year 2050 [3, 4]. Accordingly, such a major shift in the composition of Australia's population will have implications on demand for aged care services due to increased incidence and prevalence of a wide range of chronic illnesses and subsequent decreased ability to perform functional activities of daily living [5]. Additionally, the impact of population ageing will reduce the availability of service provision due to the declining health and aged care workforce [4, 6, 7]. Given this demographic shift, it is clear that aged care service providers and health professionals will need to adapt and foster innovative models of care to overcome the challenges of rising demand and decreased service capacity [8]. In 2012 the Australian Commonwealth introduced the *Living Longer Living Better* reforms, which focussed on increasing the proportion of aged care delivered in the community to reduce dependency on long-term supportive services and delaying an older person's transition to residential aged care [1, 9]. Central to these reforms, the reablement model of care has been mandated by the Australian Government through guidelines for delivery of all community based aged care [9].

Over the last two decades, reablement has emerged as a practice model in community-based home aged age [10]. The reablement model challenges the traditional model of aged care of maintenance and support, shifting to a focus which aimed at preventing further functional decline and a restoration of lost function [11–14]. A recent Delphi study defined reablement as a "person-centered and holistic approach that aims to increase or maintain a clients' independence and participation in daily and meaningful activities (at home or in the community) and to reduce their need for long-term services and related costs" [10]. Despite its increasing adoption into practice, there remains variation between, and even within, some countries regarding its conceptual understanding and implementation [15]. In Australia, the Commonwealth Government describes reablement as a model of practice aimed at supporting older people to maintain independent in activities of daily living, to improve autonomy, physical and emotional health, while reducing the need for longer term service delivery [9].

In Australia, an individual's reablement period is typically time-limited for up to 12 weeks and consists of interventions including physical training and adaptive equipment to strengthen and improve activities which the older person has defined as important [9]. Interventions are based off reablement goals which are developed collaboratively with the older person to focus on activities of daily living which will allow them to age in their usual place of residence and to participate socially. The outcomes of an older persons reablement is generally defined by the goals which they have developed in collaboration with the reablement team. As a person-centred, holistic, and strengths-based approach to care, reablement relies on an inter-disciplinary team of nursing and allied health professionals that can meet the increased complexity of older people in need of aged care services [16].

Central to the implementation of the reablement model is ensuring that community-based health professionals working with older people can effectively collaborate to ensure the best outcomes for older people [17]. Given that reablement is a shift in the delivery of aged care through doing *with*, rather than doing *for,* a lack of specific skills from health professionals in the reablement approach is not uncommon [12]. Therefore, determining and evaluating readiness for the reablement approach across the health professional workforce is important to ensure care delivered is congruent with reablement and older people's expectations. To date research into reablement practice has focussed on the physiotherapy and occupational therapy disciplines [17–19].

Whilst literature on podiatry practice in reablement is limited, evidence suggests that older people represent the largest proportion of care provided by podiatrists in Australia and their attendance at podiatrists tends to be recurrent [20, 21]. The link between foot problems in older age which reduce mobility and decrease quality of life has been well established [22–31]. Therefore, it is reasonable to assume that podiatrists are well placed to be integrated into reablement teams. For instance, fear of falling and previous falls are important predictors which can lead to older adults to avoid activities that they are still able to do [32–34]. Podiatrist delivered interventions including prescriptive exercises aimed at increased strength and balance, provision of foot orthoses, and falls prevention and footwear education have been shown to decrease the incidence of falls among older people [35].

Accordingly, the aim of this study was to explore Australian podiatrists understanding and current practice of implementing a reablement approach to older clients.

## Methods

### Study design and ethics

This was a qualitative research study using Braun and Clarke's method of reflexive thematic analysis of semi-structured interviews. Reflexive thematic analysis allowed us to identify, describe and interpret patterns (themes) which look to understand and represent the experiences, perceptions, and understandings of the participants. During data analysis, we drew on a critical realist ontology to explore podiatrists' perspectives of how the reablement model might align with their practice and their experiences when working with older people. Ethical approval was granted by the La Trobe University Human Research Ethics Committee (HEC18458).

### Participant sampling and recruitment

The participant group was conceptualised around the following parameters: podiatrists working in Australia who speak fluent English, with more than 2 years clinical experience, and have current or prior experience in working with people aged 65 years and older and who were aware of the reablement model. Participants were recruited after having completed a web-based survey which explored the influences on podiatrists' clinical practice and implementation of the reablement model. Eligible participants were then purposively selected to achieve diversity across the following characteristics: practice background (regional, rural, metropolitan), clinical setting (public sector, private sector), participants reported exposure to the reablement model [36]. Circulation of the web-based survey was through emails of podiatry networks (including the Australian Podiatry Association) and Twitter. No remuneration was advertised or offered.

### Data collection

The interviews followed a semi-structured format, a common approach to qualitative data collection [37]. A semi-structured interview schedule was utilised to enable a fluid method of data collection which would allow the interviewer to remain responsive and flexible in asking further questions about emerging topics and ideas expressed by the participant [38]. Interviews were completed between February 2019 and April 2020 via telephone by the first author with the duration ranging from 28 to 90 min. The interview guide was developed via a literature search of studies that evaluated the experience of delivering reablement from other allied health disciplines and discussion amongst both researchers. In addition, the interview guide was guided by the clinical and academic experience of both researchers. For the full interview guide see online Supplement 1. Prior to the interview, each participant had reviewed, signed, and returned a Participant Information and Consent Form. Audio recordings of the interviews were transcribed verbatim by the lead author (n = 8) and an external services provider (n = 6) which were reviewed while listening to the recordings to ensure accuracy of transcription. Data was then anonymised by removing names and the names of others mentioned in the data. Identifiable features such as the names of workplaces were also renamed.

### Data analysis

Braun and Clarke's method for reflexive thematic analysis was used to enable data analysis due to its flexibility and variability in theoretical and analytical scope [39–41]. However, it is because of this flexibility that this method has been criticised as atheoretical, lacking in clarity and rigour, and therefore makes evaluation and synthesis of research findings difficult [42]. Therefore, Braun and Clarke [43] recommend that researchers should reflect on and specify the philosophical and theoretical assumptions which have informed their thematic analysis. Braun and Clarkes process of analysis involves six-recursive-phases of: familiarisation; coding; generating initial themes; reviewing and developing themes; refining, defining, and naming themes; and writing up [39–41]. Coding was undertaken by the first author and then reviewed and reflected upon with the second author. Coded data was then used for theme development, in reference to the original transcripts, and was led by the first author in consultation with the second author to ensure a nuanced and insightful analysis which was representative of the participants experiences and views. We used NVivo 12 software to manage the data [44].

### Reflexivity statement

During data analysis, we drew on a critical realist ontology to explore podiatrists' perspectives of how the reablement model might align with their practice and their experiences when working with older people. Critical realism includes elements of both realist and constructionist paradigms, acknowledging an individual's different perspective in their understanding of reality [40, 45, 46]. We used this approach as critical realists seek to explain and critique social conditions based on an explanation of individual tendencies and causal mechanism in order to make recommendations for policy and practice [47]. During our analysis, there was a focus on both semantic (descriptive) and latent (interpretive) features of the data using predominantly inductive coding as this approach is more rooted in the data and therefore is more congruent with a critical realist perspective. Critical realism contains elements of both realist and constructionists' schools of thought, with the researcher asking the data (participants language and experience) to tell them something about the social processes that take place in a particular situation. As the aim of this study was to explore how major reform in community aged care policy affects Australian podiatrists practice, a critical realist approach provided the appropriate lens to describe and explain these conditions [47, 48].

## Results

Fourteen participants were interviewed. Twelve were female, six worked in a regional setting, and seven in community health services. Individual demographic information of the included participants has been reported in Table 1. Interview duration ranged from 28 to 90 min.

**Table 1:** Demographic details of participants

Participant	Gender	Practice setting	Metro/Regional/Rural	State
1	F	Acute hospital	Regional	VIC
2	F	Private practice	Regional	WA
3	F	Private practice/acute hospital	Metro	VIC
4	F	Community health/private practice	Rural	WA
5	F	Community health/private practice	Rural	WA
6	F	Community health	Rural	VIC
7	F	Acute hospital/community health	Regional	NSW
8	F	Community health	Rural	VIC
9	M	Community health	Regional	VIC
10	F	Sub-acute hospital, private practice	Regional	VIC
11	F	Acute hospital	Metro	NSW
12	F	Podiatry university lecturer	Metro	VIC
13	F	Community health	Metro	VIC
14	M	Acute hospital	Regional	NSW

### Overview

The results from the interviews outline complex and multi-level influences which impact on Australian podiatrists' ability to implement the reablement model. Three themes were generated from the thematic analysis process: (i) Thinking and practicing differently, (ii) Reconciling reablement practice with competing pressures, (iii) funding influences on podiatry practice and reablement.

### Themes

#### Thinking and practicing differently

This theme describes how a change in approach from participants in their encounters with older people is required in consideration of the reablement model. Most participants viewed their work with older people as assuming the role of maintaining foot health for the older person and linking this practice within the biomedical model. When participants expressed how they believed podiatrists align with the reablement approach to aged care, they explained the role of *facilitating* reablement rather than *participating* in a client's reablement process:

*I do find in podiatry reablement that we're working with, mostly, it's really probably more around maintenance. And that is maintaining something so that people - their function may not improve, but it might… (P8)*

I think podiatry's much more seen as a treatment-focused profession. (P7)

Some participants discussed that their current practices in managing older people, which involved taking control, did not align with the principles of reablement:

I think sometimes we have a tendency, [to], take things away from people and then they feel like they're not in control or they're not, they're not participating in it. (P12)

Further, participants discussed that reablement features such as short-term interventions to maintain or improve functional ability were incongruent with their current podiatry practice. Participants explained that their undergraduate training had focussed on the provision of deficit-based interventions, which led to a dependency on the podiatry clinician instead of a more strengths-based approach inherent to the reablement approach. A participant with experience in an inter-disciplinary reablement team described how they had learned some of the reablement processes and terminology from observing other allied health disciplines:

I am not sure whether we are taught about improving function. It's more about fixing the problem, then when we can't fix the problem, we take over that job for people. (P3)

*I don't think we have much of a focus on function, um, you know, maximising function for people. Once you are working in the profession, you know working with physios and OT's [Occupational Therapists] then the words you see about reablement they definitely become clear in their meaning. (P13)*

#### Reconciling reablement practice with competing pressures

Podiatrists face a range of work-related pressures which they reported as impacting on their clinical practice and ability to deliver reablement informed care. The following sub-themes describe the two most frequently described barriers to implementing the reablement model: (i) time constraints, (ii) keeping everyone happy.

##### Time constraints

Lack of time was consistently reported as a major barrier for the implementation of reablement within podiatry practice, with the additional tasks and increased time spent with individual clients challenging to integrate into their current workflows.. Participants working in private practice settings reported 20 min appointment times were insufficient time to appropriately assess a client, design a care plan, and educate on an intervention plan. Given the major shift in their clinic structure required to integrate the reablement model there was little motivation to integrate it into clinical practice. Likewise, for participants working in a service where the provision of a reablement model was a requirement, they described the additional tasks were a burden on their already busy workload:

*But to do something like [reablement] also something that takes time. And it's the care planning: when you sit down and do the care plans for people, it takes time. When you review the care plan it takes time. So, there is this big-time barrier.* (P8)

Many participants could not realistically allocate more time with individual clients to deliver reablement interventions. Some participants described that the current demand for podiatry from older people necessitates "churning through the patients, getting the waiting list sorted and that means doing things for them" (P9). Participants described addressing the challenge of heavy workloads and high service demand by having standardised workflows where their clients were passive recipients receiving care. In practice this meant doing tasks for older people rather than attempting to assess and rebuild capacity:

*I think that to teach someone to do a job that I could do for them, especially under the time my consult lasts for, then that would be a major intrusion on my time. It's easy to do just keep doing it for them. If reablement requires podiatrists training and teaching patients to perform self-care for their feet and develop skills, then yes, something like that would be a major problem.* (P3)

##### Keeping everyone happy

Meeting the demands of managers and requirements for government funding often created tensions. Some suggested the provision of sub-optimal care was a risk in order to accommodate various competing administrative demands.

*I mean you're working to the organisations policies [and] you're trying the meet the needs of what the patient wants. Then you add what the government wants you now to do this reablement thing. For a lot of patients that just doesn't work. […] There is a risk there for the patient that we do say "yes I've 'reabled' them", and that's just to make the organisation happy that they're meeting what the government wants.* (P7)

#### Funding influences on podiatry practice and reablement

Generally, podiatry services were not funded appropriately to deliver reablement interventions. Participants described the tensions between delivering time intensive reablement interventions against the overall costs of running podiatry services. Participants described how current Commonwealth government funding arrangements tended to promote a dependency model of care which conflicted with the Commonwealth's own goal to promote reablement services.

A major barrier for podiatrists to implement reablement concerns current funding arrangements which only cover the cost of the actual time spent in consultation with the patient. Options are limited to fund preventative and supportive aids (such as footwear and orthotics). As the following quote highlights, there is frustration amongst podiatrists that the prohibitive costs of preventative and supportive aids can support a cycle of dependency relying on hands-on podiatry intervention:

*It is a cycle because you see them re-present. That's my frustration because […]I want to address the underlying reason they are here in the first place. And the fact is that the money isn't there to be able to pay for it. The money pays for me and the consumables that I have in my clinic, nothing long-term for the patient. (P1)*

Many podiatrists commented on the impact of Medicare rebated Team Care Arrangements (TCA) and how this had, over time, created business models which relied on a client "churn" (P1) to "make the money and keep [the practice] going" (P12). Some podiatrists could not reconcile the economic viability of adopting a reablement model given the structure of podiatry clinics:

*It will be a bit harder to implement in the private setting to take their focus away from, from the financial aspect of things. Taking time to do reablement with a patient will limit the amount of patients that a podiatrist could see in a day, and that will reduce their revenue, and that would be a huge barrier to doing reablement type care in those settings. (P9)*

Across the interviews, there was a sense that the expansion and bulk billing of the Medicare funded TCA had masked the cost of care provision which many felt devalued their care and decreased engagement from clients. This had the unintended consequence of increasing a client's dependency on podiatry services and, in turn, led to client's adopting a more passive role in their care which is incongruent with the aims of the reablement model:

*The [TCAs] have definitely made people more dependent. Well, it is the same with anything as you get older. If you don't use it, you lose it. So, if people are really trying hard to bend down to cut their toenails and they can, then they are going to keep doing that. But if they find that somebody else can do it for them, and they are not trying to bend down, then they probably are going to lose that. (P2)*

## Discussion

The aim of this study was to explore Australian podiatrists understanding and current practice of implementing a reablement approach when working with older people. The findings presented here suggest that there are complex multi-level influences which have impact podiatrists practice and attitudes towards their clinical practice when working with older people and their ability to apply the reablement model. Three themes characterised podiatrists' views and attitudes towards reablement: *Thinking and practicing differently, Reconciling reablement practice with competing pressures, Funding influences on podiatry practice and reablement*.

Overwhelmingly, participants viewed reablement as a major shift in how they delivered care to older people. Participants mostly described their practice in aged care as within the biomedical model, and that this was strongly influenced by current funding models whilst also acknowledging a general lack in knowledge and skills to deliver reablement. Participants with greater experience working within the reablement model explained how other disciplines such as physiotherapy and occupational therapy had been influential in their education. For others, a lack of understanding and ability to apply reablement was attributed to their training as a podiatrist. Most participants linked a clients need to see a podiatrist with a medical condition. The implications for this approach to care has been explored by Boden [49], who interviewed older people in England receiving podiatry care and reported that through the podiatrist's medicalisation of footcare, older people acquiesce into the *patient role,* becoming passive recipients of care leading to increased dependency on podiatrists.

A change in societal beliefs and service preferences has been identified as a strong external force on changing professional boundaries [50]. Gill and Cameron [51] explored Australian baby boomer expectations for aged care services and reported that specific changes to the industry which focus on truly empowering older people to self-determine and self-manage. Boden [49] identified that by approaching care through the biomedical model, identifying medical conditions, and linking these to a client's foot health, podiatrists will not fully appreciate their clients' expectations and needs. In contrast, application of the reablement model by podiatrists could facilitate their assessment of how older people manage their foot health, with a focus on recourses that will support care that is focussed on enhancing independence.

Working with older people is a significant part of podiatry practice and previously identified as a major source of occupational stress amongst Australian podiatrists [21, 52]. Tinley [52] reported that Australian podiatrists describe their work with older people as repetitive and insufficiently challenging with perceived limited clinical gains, akin to a palliative approach to care, and that this contributed to stress. Care delivered within a reablement focus can have a positive impact on health professionals, increasing their job satisfaction, and reducing attrition compared to working within more traditional models of care [53].

Population ageing will mean that older people are increasingly in need of health and aged care services, supporting a strong rationale for a change in focus to more progressive care models that address functional decline and shift attitudes of aged care practice. However, the appeal for increased focus of aged care podiatry development have been documented for almost two decades [52, 54], yet the response from participants in our study suggests that aged care practice training continues to be limited and remains a low status area within the profession.

Participants in the current study also highlighted a lack of appropriate funding arrangements which would support the implementation of reablement in podiatry practice. Private sectors participants were particularly impacted and had concerns that the limitations of Medicare TCA had inadvertently increased older people's dependency on podiatry services. Previous research has identified issues in allied health practice where the TCA only funds the consultation fee and none of the associated supplies, aids and equipment required for more effective management of older people [55, 56]. Consequently, these associated costs must be financed by the client, whose socioeconomic status impacts their ability to pay and the subsequent uptake of recommendations by the podiatrist [56]. Other concerns raised about the effects of Medicare TCA on practice related to the inadequacy of the rebate to maintain a viable business [55–57]. This study also identified that low levels of rebate resulted in a tendency to "churn" clients through to ensure sufficient income to sustain a financially viable practice.

### Strengths and limitations

A key strength of the current study was the use of in-depth interviews with practicing podiatrists. Whilst this limits the number of participants, the data attained is rich and provides insights not possible with quantitative methods. A benefit of individual interviews over focus groups was the confidentiality provided meaning participants may feel more comfortable discussing their personal experiences. Using purposeful sampling podiatrists were recruited from across clinical settings with experience from 4 years to almost 30 years. Telephone interviews offered the advantage of recruiting from diverse geographical locations.

As with all studies, limitations exist. The study population was biased in relation to gender and is not a true reflection of the current podiatry workforce in Australia. Telephone interviews, whilst also discussed as a strength, can potentially make developing a rapport with the participants more difficult and this could limit levels of engagement with the participant [58]. Further, telephone interviews do not allow us to observe visual cues which could assist and influence with the interpretation of the data. Finally, as reablement is an emergent model of community aged care in Australia, experience of working with the model was varied. Whilst some participants were very familiar with reablement, others were less familiar. We addressed this issue by providing both written and verbal information on the topic (both prior and during the interview), however low levels of experience may have meant in some interviews in-depth discussion of the application of reablement was limited.

## Conclusion

Reablement is an increasingly adopted model of aged care practice within Australia. This study has provided an understanding of some of the current perceived barriers which may impact on its implementation within podiatrists practice when working with older people. The findings suggest that some podiatrists lack knowledge and skills in the application of biopsychosocial informed models of care, that there is limited time in a podiatry consultation, and that current funding arrangements are inadequate. It is apparent from our study that systemic, and seemingly intractable challenges, appear to impact the uptake of the reablement model in Australian podiatrist's practice. Therefore, in addition to increased training and awareness of more holistic approaches to care, major structural changes, including reform to funding structures which would facilitate reablement practice are needed to facilitate Australian podiatrists adopting the reablement model into their clinical practice.

## Data Availability

Please contact author for data requests.

## Abbreviations

GDP: Gross domestic product
TCA: Team Care Arrangements
OT: Occupational Therapist
